# Supplementary material for: Indexcov: fast coverage quality control for whole-genome sequencing
Source: Gigascience. 2017 Sep 18;6(11):1–6. doi: 10.1093/gigascience/gix090 (PMC5737511; doi:10.1093/gigascience/gix090)
Supplement: Supplementary Figure [file gix090_supp.docx]

## **Indexcov: fast coverage quality control for whole-genome sequencing**

Brent S. Pedersen^1,3,^**^+^**, Ryan L. Collins^4,6,7^, Michael E. Talkowski^4,5,6,7^, Aaron R. Quinlan^1,2,3,^**^+^**

1. Department of Human Genetics, University of Utah, Salt Lake City, UT

2. Department of Biomedical Informatics, University of Utah, Salt Lake City, UT

3. USTAR Center for Genetic Discovery, University of Utah, Salt Lake City, UT

4. Center for Genomic Medicine, Massachusetts General Hospital, Boston, MA

5. Department of Neurology, Harvard Medical School, Boston, MA

6. Program in Medical and Population Genetics and Stanley Center for Psychiatric Research, Broad Institute, Cambridge, MA

7. Program in Bioinformatics and Integrative Genomics, Division of Medical Sciences, Harvard Medical School, Boston, MA.


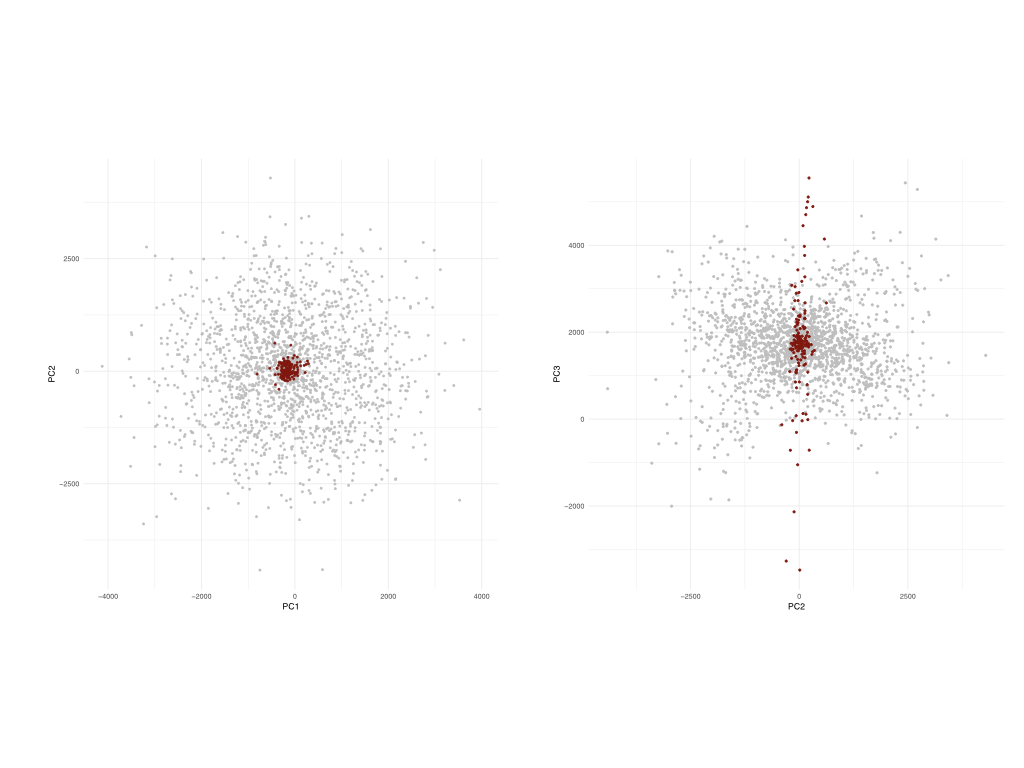


**Supplemental Figure 1.** For the 2,076 samples from the Simons Simplex Autism cohort, we plot the first 2 principal components. Samples that were prepared with a PCR-free method are shown in red, with the remaining samples in gray. We see that the samples that had a PCR step have a much greater spread in the principal component values due to the greater variation in genomic coverage resulting from PCR amplification prior to sequencing[^11^](https://paperpile.com/c/sHS8t8/4uIUa).
