# Supplementary material for: Indexcov: fast coverage quality control for whole-genome sequencing
Source: Gigascience. 2017 Sep 18;6(11):1–6. doi: 10.1093/gigascience/gix090 (PMC5737511; doi:10.1093/gigascience/gix090)
Supplement: Response-to-Reviewer-Comments_Original-Submission.pdf [file gix090_response-to-reviewer-comments_original-submission.pdf]

Author's Response To Reviewer Comments

Reviewer reports:
Reviewer #1: In their paper , the authors use the BAM index (bai) to get a rough idea of the presence of CNV . The idea is really original and the implementation is open-source. I don't have much to say about the paper itself: it's clear, with convincing examples.

But , the main point is that I've used this software on a set of more than 500 WGS data: the processing was quick and within a few minutes we have (probably) detected a large somatic deletion in one of our sample that was unknown to us.

On my side, the paper can be accepted without revision as is. If you have the opportunity to update the paper, I'd like to hear that the estimation of the number of reads is not only biased by the number of supplementary reads but also by the duplicate/MAQ=0/failing-QC etc…

>>
We examined this for bins that were different between samtools and indexcov and found no conclusive pattern for tags (splitters), duplicates, or other flags. See response to reviewer 2 below.
>>

Furthermore it's not clear to me if how this software would work if the majority of the reads were long reads (>17kb) ?

>>
Indexcov works best with shorter reads. We have run this on nanopore data with 7KB median read-length and the sampling error due to having fewer reads per bin results in highly variable coverage estimates to the point of being not useful.
>>

Pierre Lindenbaum

Reviewer #2: The authors describe a tool, goleft indexcov, to estimate the depth of coverage of a BAM file using only the BAM index (.bai) file. The tool is much faster than samtools depth. The authors report that the estimate is reasonably accurate, being within 5% of the true value 97% of the time. I believe this tool is quite useful, due to its notable speed and lack of similar QC tools that summarize depth of coverage statistics across samples.

My best regards,
Shaun Jackman

Introduction

Is indexcov expected to work with long reads? How are alignments that are longer than the BAM block size of 16,384 bp treated? Does an alignment of, for example, 20 kbp that spans three bins count toward one of those bins, or all three? If one bin, which?

>>
The read spanning multiple bins would only contribute to the first bin. See also the response to Pierre above about long reads.
>>

Is the size in bytes of each bin before or after compression, and would a compressed BAM file give different results than an uncompressed BAM file?

>>
It is the size on disk, so if compressed BAM it is based on compressed size, otherwise uncompressed.
>>

Results

Please provide a correlation statistic of indexcov and samtools, such as Pearson or Spearman.

>>
The Pearson correlation is 0.81, the spearman correlation is: 0.55. The spearman correlation increases to > 0.9 when we only evaluate regions that do not overlap a low complexity region, however, this removes a lot of regions from the comparison. Of the bins that did not overlap LCRs, only 5 had a difference. One of those preceded a N-masked region in the genome where indexcov over-estimated the coverage. Indexcov also overestimated coverage in the other 4 regions. We did not conclude anything about these bins such as an increase of splitters, or duplicates. It could be, for example, that the genomic sequence in these regions is generally less compressible, so the data in the bams is less compressed and indexcov over-estimates the coverage.

Our aim is to show that this works most of the time, not that it is a replacement for depth based on the actual alignments, we have added a note to that effect in the conclusion and made wording changes in the text to indicate that this is to supplement per-alignment coverage methods.
>>

> Whereas this analysis required ~8 minutes of CPU time for samtools on chromosome 1,
67 indexcov completed the analysis in 1 second for the entire genome.

Please report the CPU time required for `samtools depth` on the entire genome. Giving the CPU time for `bedtools genomecov -ibam` would further strengthen the case.

>>
bedtools 103m29s
We have updated the text to indicate that samtools took ~61 minutes for the whole genome. Bedtools took ~104 minutes. This is now in the manuscript.
>>

Figure 1

What is the maximum value of indexcov - samtools, and is the axis truncated? If so it should be indicated in the caption.
>>
15196 regions. 420 (2.76%) of those not shown in plot. The maximum difference is > 10.
>>

A scatter plot or heat map of indexcov vs. samtools would be helpful if space permits.

>>
Most of the data is centered at 1,1 so the correlation is not very high and the scatter plot just shows a dense cloud of points at 1,1. We have attempted to show that on the large scale, indexcov gives a rather good estimate of coverage, without overstating. The histogram of the delta seems a good way to show, on average how far off the estimate is likely to be.
>>

Figure 3

Labeling the clusters with XX, XY, XXY, XYY would add clarity.

>>
Agreed - done.
>>

Adding transparency (alpha) to the data points would help mitigate the overplotting.

>>
The overplotting generally occurs at X=1, Y=1 and X=2, Y=0, corresponding to the 2 sexes. Those points are generally less interesting than the points that fall outside of those clusters. That said, we will consider adding alpha to a future release.
>>

Identifying Aberrant Genome-Wide Coverage Profiles

The parameter 0.15 may not be necessary if the tool were to report the number of outliers, defined as the number of bins with coverage less than Q1 - 1.5 * (Q3 - Q1). The range (0.85, 1.15) could similarly be based on the IQR (Q3 - Q1). Just a thought. No change to the manuscript is necessary.

>>
Given a sample with a large variability, which is what we are aiming to detect, the IQR would be increased as well. While the current metric seems and is arbitrary, we have found that it is effective at detecting the signal we are looking for.
>>

Figure 4

Are the PCR-free library preps less variable in coverage? Please use colour to distinguish the PCR-free data points.

>>
We have updated the figure to color the PCR free and PCR groups differently.
>>

Speed & Scaling

> we use only 1 byte per scaled coverage value for each sample that we accumulate

How is the coverage encoded in one byte, fixed-point binary or minifloat?

>>
The estimated coverage is scaled to a value between 0 and 255 so it will fit in a byte. It is later converted to a larger value, but this is after other memory has been cleared.
>>
