# Supplementary material for: Indexcov: fast coverage quality control for whole-genome sequencing
Source: Gigascience. 2017 Sep 18;6(11):1–6. doi: 10.1093/gigascience/gix090 (PMC5737511; doi:10.1093/gigascience/gix090)
Supplement: Reviewer-1-Report-(Original-Submission).pdf [file gix090_reviewer-1-report-(original-submission).pdf]

Reviewer Report

Title: Indexcov: fast coverage quality control for whole-genome sequencing

Version: Original Submission **Date:** 7/20/2017

Reviewer name: Pierre Lindenbaum

Reviewer Comments to Author:

In their paper , the authors use the BAM index (bai) to get a rough idea of the presence of CNV . The idea is really original and the implementation is open-source. I don't have much to say about the paper itself: it's clear, with convincing examples.But , the main point is that I've used this software on a set of more than 500 WGS data: the processing was quick and within a few minutes we have (probably) detected a large somatic deletion in one of our sample that was unknown to us.On my side, the paper can be accepted without revision as is. If you have the opportunity to update the paper, I'd like to hear that the estimation of the number of reads is not only biased by the number of supplementary reads but also by the duplicate/MAQ=0/failing-QC etc... Furthermore it's not clear to me if how this software would work if the majority of the reads were long reads (>17kb) ?Pierre Lindenbaum

Level of Interest

Please indicate how interesting you found the manuscript: An article of limited interest

Quality of Written English

Please indicate the quality of language in the manuscript: Acceptable

Declaration of Competing Interests

Please complete a declaration of competing interests, considering the following questions:

- Have you in the past five years received reimbursements, fees, funding, or salary from an organisation that may in any way gain or lose financially from the publication of this manuscript, either now or in the future?
- Do you hold any stocks or shares in an organisation that may in any way gain or lose financially from the publication of this manuscript, either now or in the future?
- Do you hold or are you currently applying for any patents relating to the content of the manuscript?
- Have you received reimbursements, fees, funding, or salary from an organization that holds or has applied for patents relating to the content of the manuscript?
- Do you have any other financial competing interests?
- Do you have any non-financial competing interests in relation to this paper?

If you can answer no to all of the above, write 'I declare that I have no competing interests' below. If your reply is yes to any, please give details below.

I declare that I have no competing interests

I agree to the open peer review policy of the journal. I understand that my name will be included on my report to the authors and, if the manuscript is accepted for publication, my named report including any attachments I upload will be posted on the website along with the authors' responses. I agree for my report to be made available under an Open Access Creative Commons CC-BY license (http://creativecommons.org/licenses/by/4.0/). I understand that any comments which I do not wish to be included in my named report can be included as confidential comments to the editors, which will not be published.

I agree to the open peer review policy of the journal
