# Supplementary material for: Indexcov: fast coverage quality control for whole-genome sequencing
Source: Gigascience. 2017 Sep 18;6(11):1–6. doi: 10.1093/gigascience/gix090 (PMC5737511; doi:10.1093/gigascience/gix090)
Supplement: Reviewer-2-Report-(Original-Submission).pdf [file gix090_reviewer-2-report-(original-submission).pdf]

Reviewer Report

Title: Indexcov: fast coverage quality control for whole-genome sequencing

Version: Original Submission **Date:** 7/31/2017

Reviewer name: Shaun Jackman

Reviewer Comments to Author:

The authors describe a tool, goleft indexcov, to estimate the depth of coverage of a BAM file using only the BAM index (.bai) file. The tool is much faster than samtools depth. The authors report that the estimate is reasonably accurate, being within 5% of the true value 97% of the time. I believe this tool is quite useful, due to its notable speed and lack of similar QC tools that summarize depth of coverage statistics across samples.My best regards,Shaun Jackman# IntroductionIs indexcov expected to work with long reads? How are alignments that are longer than the BAM block size of 16,384 bp treated? Does an alignment of, for example, 20 kbp that spans three bins count toward one of those bins, or all three? If one bin, which?
Is the size in bytes of each bin before or after compression, and would a compressed BAM file give different results than an uncompressed BAM file?# ResultsPlease provide a correlation statistic of indexcov and samtools, such as Pearson or Spearman.> Whereas this analysis required ~8 minutes of CPU time for samtools on chromosome 1,67 indexcov completed the analysis in 1 second for the entire genome.Please report the CPU time required for `samtools depth` on the entire genome. Giving the CPU time for `bedtools genomecov -ibam` would further strengthen the case.# Figure 1What is the maximum value of indexcov - samtools, and is the axis truncated? If so it should be indicated in the caption.A scatter plot or heat map of indexcov vs. samtools would be helpful if space permits.# Figure 3Labeling the clusters with XX, XY, XXY, XYY would add clarity.Adding transparency (alpha) to the data points would help mitigate the overplotting.# Identifying Aberrant Genome-Wide Coverage ProfilesThe parameter 0.15 may not be necessary if the tool were to report the number of outliers, defined as the number of bins with coverage less than Q1 - 1.5 * (Q3 - Q1). The range (0.85, 1.15) could similarly be based on the IQR (Q3 - Q1). Just a thought. No change to the manuscript is necessary.# Figure 4Are the PCR-free library preps less variable in coverage? Please use colour to distinguish the PCR-free data points.# Speed & Scaling> we use only 1 byte per scaled coverage value for each sample that we accumulateHow is the coverage encoded in one byte, fixed-point binary or minifloat?

Level of Interest

Please indicate how interesting you found the manuscript: An article of importance in its field

Quality of Written English

Please indicate the quality of language in the manuscript: Acceptable

Declaration of Competing Interests

Please complete a declaration of competing interests, considering the following questions:

- Have you in the past five years received reimbursements, fees, funding, or salary from an organisation that may in any way gain or lose financially from the publication of this manuscript, either now or in the future?
- Do you hold any stocks or shares in an organisation that may in any way gain or lose financially from the publication of this manuscript, either now or in the future?
- Do you hold or are you currently applying for any patents relating to the content of the manuscript?
- Have you received reimbursements, fees, funding, or salary from an organization that holds or has applied for patents relating to the content of the manuscript?
- Do you have any other financial competing interests?
- Do you have any non-financial competing interests in relation to this paper?

If you can answer no to all of the above, write 'I declare that I have no competing interests' below. If your reply is yes to any, please give details below.

I declare that I have no competing interests'.

I agree to the open peer review policy of the journal. I understand that my name will be included on my report to the authors and, if the manuscript is accepted for publication, my named report including any attachments I upload will be posted on the website along with the authors' responses. I agree for my report to be made available under an Open Access Creative Commons CC-BY license (http://creativecommons.org/licenses/by/4.0/). I understand that any comments which I do not wish to be included in my named report can be included as confidential comments to the editors, which will not be published.

I agree to the open peer review policy of the journal
